# The Table to Tablet (T2T) Speech and Language Therapy Software Development Roadmap

**DOI:** 10.2196/11596

**Published:** 2019-01-30

**Authors:** Luis MT Jesus, Joaquim Santos, Joana Martinez

**Affiliations:** 1 University of Aveiro Aveiro Portugal

**Keywords:** software, tablets, children, speech sound disorders, design-based research

## Abstract

**Background:**

Few studies have analyzed gains in using computers in speech and language therapy interventions for children with speech and/or language disorders when compared to a control group, but virtual tutors and computer-based visual feedback have been gaining interest in the literature. Previous systematic reviews mainly focused on development technological details of computer-based speech training systems or the potential of integrating mobile technology into education and rehabilitation, but recent systematic reviews have also evaluated the efficacy of computer-based speech and language therapy for children and how digital technology can support different activities, at school or elsewhere.

**Objective:**

This study aimed to analyze a continuous communication and joint team approach to develop solutions focused on the real needs of end users, which digitally emulate reliable and validated physical intervention materials for children with speech sound disorders (SSD).

**Methods:**

The Table to Tablet (T2T) software was developed using a design-based research methodology, which included four phases: activities development; ethnographic pretesting with a sample from the target population; software development; and beta-testing. The technology used to develop the software, the method used to ensure satisfaction and replay ability of the intervention materials, and results from the ethnographic and beta-testing phases are presented.

**Results:**

Nineteen activities were developed during the first phase, which were then tested, with 7 service users, using a physical prototype. The beta-test approach included extensive testing and reformulation, supported by direct, nonparticipant observation and data collection using a questionnaire designed for children. Feedback was used to improve the software and interaction with users.

**Conclusions:**

The use of T2T-based intervention programmes by speech and language therapists (SLTs) will allow these professionals to make a better and more effective communication intervention, based on proven methodologies, that coexists in a structured physical and a digital version. These versions provide a full, 6-week intervention program, with minimal effort in preparing the session by the SLTs while delivering a very consistent intervention, with high replay value. A continuous communication and joint team approach was beneficial to the project and to the development of a solution focused on the real needs of SLTs and children with SSD. All problems were approached as a team with different skills and expertise, which minimized errors (eg, the developer making choices that would save him from spending time doing something that would not be used) and time spent. To add to this, the importance of integrating the end users as testers and collecting their opinions and actions per session allowed the production of better-targeted activities.

**Trial Registration:**

ClinicalTrials.gov NCT02490826; https://clinicaltrials.gov/ct2/show/NCT02490826

## Introduction

### Background

Children with speech sound disorders (SSD) represent 40% to 90% of pediatric caseloads [1-3]. They present gaps in their speech sound systems that might cause difficulties in producing or understanding speech sounds [4,5]. They can have substitution errors, syllable structure errors, speech sounds distortions, and atypical prosody [4].

A previous paper [2] explored through a Web-based survey the most common intervention strategies used by speech and language therapists (SLTs) to treat children with SSD, concluding that these included auditory bombardment, hearing and discriminating, grapheme-phoneme correspondence, phoneme identity, segmentation, blending, rhyme, and phoneme manipulation. On the basis of these results, a randomized controlled study was conducted [6] to test the efficiency and efficacy of using a combination of these intervention strategies. This approach (a combination of expressive phonological tasks, phonological awareness, and listening and discrimination activities) [6], based on a physical set of activities (tabletop), was shown to be an effective integrated method of treating children with SSD.

Few studies [7-11] analyzed gains in using computers in speech and language therapy intervention for children with speech and language disorders, when compared with a control group, but virtual tutors and computer-based visual feedback have been gaining interest in the literature [12-18]. Previous systematic reviews have mainly focused on the development of technological details of computer-based speech training systems [14,19] or the potential of integrating mobile technology into education and rehabilitation [16,20]; however, recent systematic reviews [21] have also evaluated the efficacy of computer-based speech and language therapy for children with SSD and how digital technology can support different activities, at school or elsewhere [22]. Furlong et al [21] concluded that there were only 14 studies, with small sample sizes and study qualities from moderate to low. They highlighted the importance of collaboration between software developers, designers, and SLTs in developing computer-based interventions and recognized the “rising popularity of mobile applications” [21]. They also concluded that “it is not possible to determine whether results are attributable to intervention or maturation” [21] without a control group.

This paper builds on this previous research [2,6,23,24], by digitally emulating the previously described tabletop approach, which was shown [6] to be a valid framework of intervention materials for children with SSD.

### The Table to Tablet Software Intervention Framework

**Figure 1:**
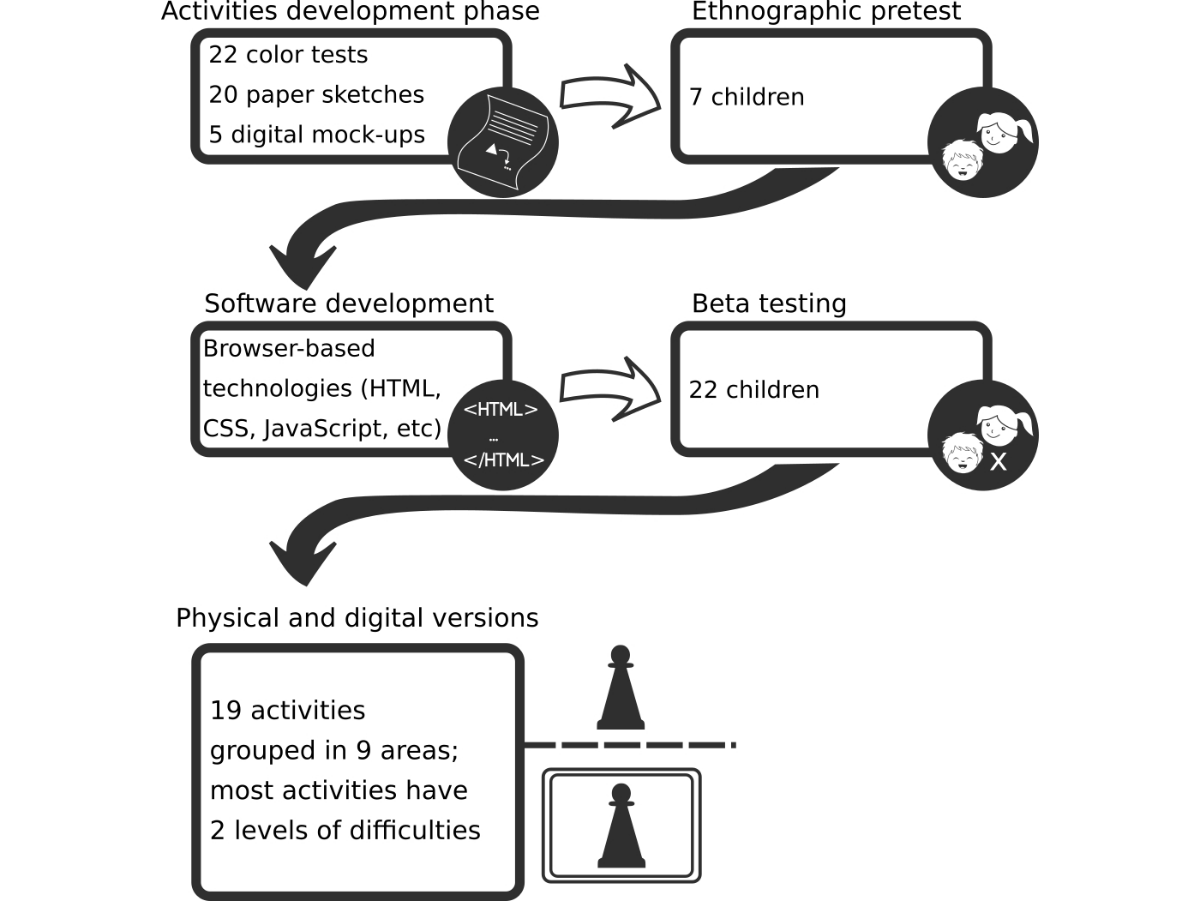
The Table to Table to Tablet (T2T) development roadmap. CSS: cascading style sheets.

This paper details the development roadmap of the digital version of a novel intervention framework for SLTs named *Table to Tablet* (*T2T*) and how it digitally emulates its physical counterpart, the technology used to develop the software, the methods used to ensure consistency of the intervention materials, and the feedback and results from an ethnographic approach and beta testing. The development framework and the main outcomes of each stage are highlighted in Figure 1.

Our long-term goals are to improve interaction and functionality of software, with more languages offered, different activities to address various areas of speech and language therapy or language acquisition, creation of an easy-to-use database that can be accessed by STLs, and developing digital homework for the children. Regular homework is recommended for maximizing progress [25]. Since the *T2T* software aims to emulate a physical framework of intervention materials for children with SSD, SLTs will be able to seamlessly swap between physical and digital materials, without compromising the efficiency and efficacy of their intervention strategies.

To better understand the market needs, a competitor analysis [26] was conducted. Results showed that there are some off-the-shelf tabletop (eg, board games or physical objects) and digital materials that can be used by SLTs to support the intervention, but they are not widely distributed in some countries, and more importantly, their efficiency has not been tested. Bowen [4] concludes that there is a gap between the technological development and the increase in evidence that technology can enhance intervention outcomes.

However, children nowadays live in an environment surrounded by electronic devices, computers, mobile phones smartphones, and other technologies that change their interactions and learning preferences [27]. To adapt to this new paradigm, SLTs need to innovate and expand the strategies and activities to better suit the interests of today’s children. The use of software is one commonly adopted solution [28]. A computer game–based approach in teaching and learning can be an effective tool to promote and enhance learning experiences and children’s motivation [29].

SLTs mostly provide individual treatment to children, and the intervention can occur in different contexts: hospitals, clinics, kindergartens, or schools. This usually implies carrying large quantities of intervention materials (such as board games, puppets, and other materials) or alternatively [4], just carry 1 device loaded with specific apps, targeted at SLTs’ needs. These apps have, however, varying efficacies [4,21]. The intervention usually takes place once a week, over a period exceeding 6 months [2].

Game activities help the child develop various skills such as visual intelligence, problem solving, and creativity [30]. Another advantage is that intervening with the aid of a computer can be disguised as *gaming time*, thus presenting additional opportunities for learning [31]. The use of these activities provides a selective and individualized therapeutic approach, while being very motivating for children and even for SLTs [32].

### Computer-Based Speech and Language Therapy Technological Requirements

Tablets are gaining ground over laptops/desktops because of their tactile nature, which is closer to user reality [33,34], and their mobility, dissemination, and growing popularity [35,36]. However, these devices have different operating systems or variations of them, screen sizes, and resolutions. A “write once, run anywhere” (slogan coined by *Sun Microsystems*) mind-set is, therefore, deemed necessary when developing software for tablets. An 8-inch tablet screen size allows the device to be easily held by a child, while having the necessary dimension as to not strain the user’s eyes. Moreover, the current worldwide market share of small size (7-9 inch) screen tablets is by far the largest (around 55% according to a study [37]). Tablet-based intervention activities need to be run online or offline, the latter being a necessity due to the variety of locations where a speech and language therapy session might occur (ie, no guaranteed internet or cell phone connectivity at sessions or at the user’s household).

The requirements of T2T software were to work nearly identically across all platforms currently available and has the following advantages over more traditional table-based therapy materials: more durable (no wear and tear), reduced preparation time, better organization (all activities and images in one place), easier to carry, and cheaper than a physical version.

### Purpose

The purpose of this paper is to document the *T2T* software development roadmap and its implicit joint-team approach. Early studies [6] on the development of intervention approaches for children with SSD were mainly conducted by SLTs. However, the technology evolution and the needs from the users are constantly changing; therefore, multidisciplinary teams are needed [21]. We aim to assess the outcomes of having such teams (as described in the study by Furlong et al [21]) involved in the development and testing processes.

Using a design-based research (DBR) methodology, 4 phases of development and joint collaboration were defined: activities development, ethnographic pretest (with sample from target population), software development, and beta test. Choices and technical aspects behind the *T2T* software, the technology used to develop the software, the method used to ensure satisfaction and replay ability of the intervention materials, and the results of the ethnographic phase and beta test phase are also included.

## Methods

### The Design-Based Research (DBR) Method

There are several software development methods, from the most traditional approach of the waterfall method to the newest Scrum approach, all with their pros and cons. Similarly, there are several design methods, with different focus, advantages, and disadvantages. However, their value and significance has to be considered in the context of this particular project’s objective, that is, the development of a speech and language therapy intervention tool for children with SSD, with a physical and a digital stand-alone component, based on a multidisciplinary team with very different backgrounds. Both the physical and the digital components had to mimic each other perfectly to avoid any skewing factor. We, therefore, sought a method that is well suited for the creation of prototypes.

DBR, the chosen methodology, is capable of producing 2 nonexclusive outputs [38]: the theoretical and the practical outcomes. The DBR model starts from a complex and real problem (in this case children’s SSD and the needs of a digital validated intervention software) and follows an iterative process going back and forth between developing, testing, and rethinking. Therefore, there is a practical outcome (the *T2T* software) and a theoretical contribution (eg, a previous publication [39] focusing on the impact of the service delivery during this project and the current paper produced during this project). The constant iteration and user feedback gathered using the DBR method facilitates an experience akin to the users being cocreators and allows for faster prototype development and more tests.

As previously mentioned, before software development, data to inform the design of intervention materials were collected from end users (SLTs), through an online survey [2]. A combination of the most common intervention strategies reported by the SLTs that participated in our previous study [2], were later [6] shown to be effective when presented in a physical format (tabletop). An emulation of these activities (previously tested in the study by Lousada et al [6]) was the basis for the development of the *T2T* software that went through 4 distinct phases: activities development; ethnographic pretesting with a sample from the target population; software development; and beta testing. Since the DBR method was being used, these 4 distinct phases were iterated more than once, until the final product was deemed stable/finalized. The software development and beta testing phases, in particular, produced several iterations.

### Activities Development Phase

During this phase, the research and development team (a speech scientist, 2 SLTs, 2 software developers, and a designer) analyzed traditional/conventional tabletop activities, materials and theories reported in the study by Lousada et al [6], and discussed how they could be implemented in both environments (physical and software). The word “activities” refers to the exercises done by children, with the direct supervision of an SLT that are the basis of interventions for children with SSD. They usually consist of traditional games, for example, a puzzle adapted to achieve a certain therapy goal. In the case of a puzzle, a certain target word can be elicited by showing images related to it and if the child is able to correctly produce the word, he/she can place a piece in the puzzle.

One important issue the team had to tackle was the screen dimensions versus real-world tabletop dimensions. Everything had to be seamless and consistent across media. Low- and high-fidelity prototyping was used to develop 22 color tests, approximately 20 paper sketches, and 5 digital mock-ups. This planning and mock-up building phase allowed the sketching/drafting of several activities for the various intervention areas.

The *T2T* intervention software includes 19 different activities, grouped in 9 areas, namely auditory bombardment, hearing and discriminating, grapheme-phoneme correspondence, phoneme identity, segmentation, blending, rhyme, phoneme manipulation, and generalization task.

The activities combine tasks of phonological expression, phonological awareness, listening, and discrimination that have been shown to be an effective integrated method to remediate SSD [6,23]. Most of the activities have 2 levels of difficulty differentiated by the inclusion or absence of the written word. For each problem addressed, a list of 15 words was selected. Furthermore, as facilitator sounds, 5 contrasting sounds (easily produced by the children) were used, and 10 words where these sounds occur were selected. Furthermore, 18 short stories that used 20 words with the target sound were also created.

Each target word was illustrated by a professional designer, resulting in a total of 335 illustrations. A specific background image was also created (by the same designer) for each short story. The lettering used for all the materials used the Verdana font due to previously published research evidence [40,41] showing that children read and search texts more quickly using this font. Additional graphic materials (for the graphic user interface) were also developed and over 950 sound productions were recorded.

### Ethnographic Pretest

Ethnography is a qualitative research method used in human-centered design [42,43] to expose opinions and concepts from groups of people [44]. In this phase, the physical tabletop materials were built and pretested in a sample of the target population. The team opted to use the physical materials first because they would allow them to determine the actual needs, what content to include, and what data to gather. The pretest sample consisted of 7 children, 4 girls and 3 boys, with an age range from 48 to 67 months (mean 57.5 months), all diagnosed with SSD. All ethical procedures were ensured, and informed consent was collected from all carers before any data collection. The testing consisted of 6 sessions based on the materials and predefined activities, with constant monitoring and feedback gathering by an SLT acting as a participant observer (the SLT would record notes during or after session but also engage in the activities with the child) as befits the ethnographic approach. This is particularly difficult in children with SSD because they might struggle to communicate, and sometimes, the speech they produce is difficult to understand [4].

To ensure consistency, validity, minimize errors, and man power costs of the software developed [45], 2 testing periods were conducted: alpha testing, during this pretest ethnographic phase and beta testing on our fourth development phase. An alpha test is the process of testing for the first time, in-house, newly developed hardware or software [46]. In the *T2T* case, the alpha test was conducted using a physical version to ascertain the feasibility of our activities and to ensure that the SLTs’ needs were correctly interpreted. The testers were the research and development team (6 members) and a sample from the target population (7 users), during our second phase. These 7 testers were from a kindergarten in the university campus, minimizing the time spent on trips and allowing for greater control of all variables involved in the sessions.

The feedback and observation allowed the team to modify or even create new activities and validate the intervention materials and techniques. With that information, the team was able to create simplified flowcharts for the software, and the first versions of different tablet-based activities were designed.

### Software Development

This development phase started concurrently with the ethnographic pretest phase. To be able to meet the requirements and be platform agnostic, the hybrid app approach was used. Browser-based technologies were used to develop the *T2T* framework so that it would be scalable, faster to develop, and cost/time effective. Hybrid apps are primarily built using HTML, CSS, and JavaScript, which is then wrapped inside a thin native container that provides access to native platform features [47]. The outcome is an .apk file that can be published in an *App Store* and the user can easily install.

The *T2T* software extensively uses HTML5, CSS, JSON, and JavaScript (Phaser framework) [48] as the building blocks of activities. To ensure cross-platform mobile versatility, we used the command line Apache Cordova as a code wrapper for the mobile environment with the Crosswalk plugin to enable cutting-edge HTML5 browser features on the devices. At the moment, user choices regarding sounds and activities are stored and retrieved using HTML5 local storage. In future versions, we plan to use a custom nonrelational database (such as MongoDB) that will store these data and other deemed necessary for the STLs’ appraisal of adherence to therapy [49] whenever the device connects to the internet.

**Figure 2:**
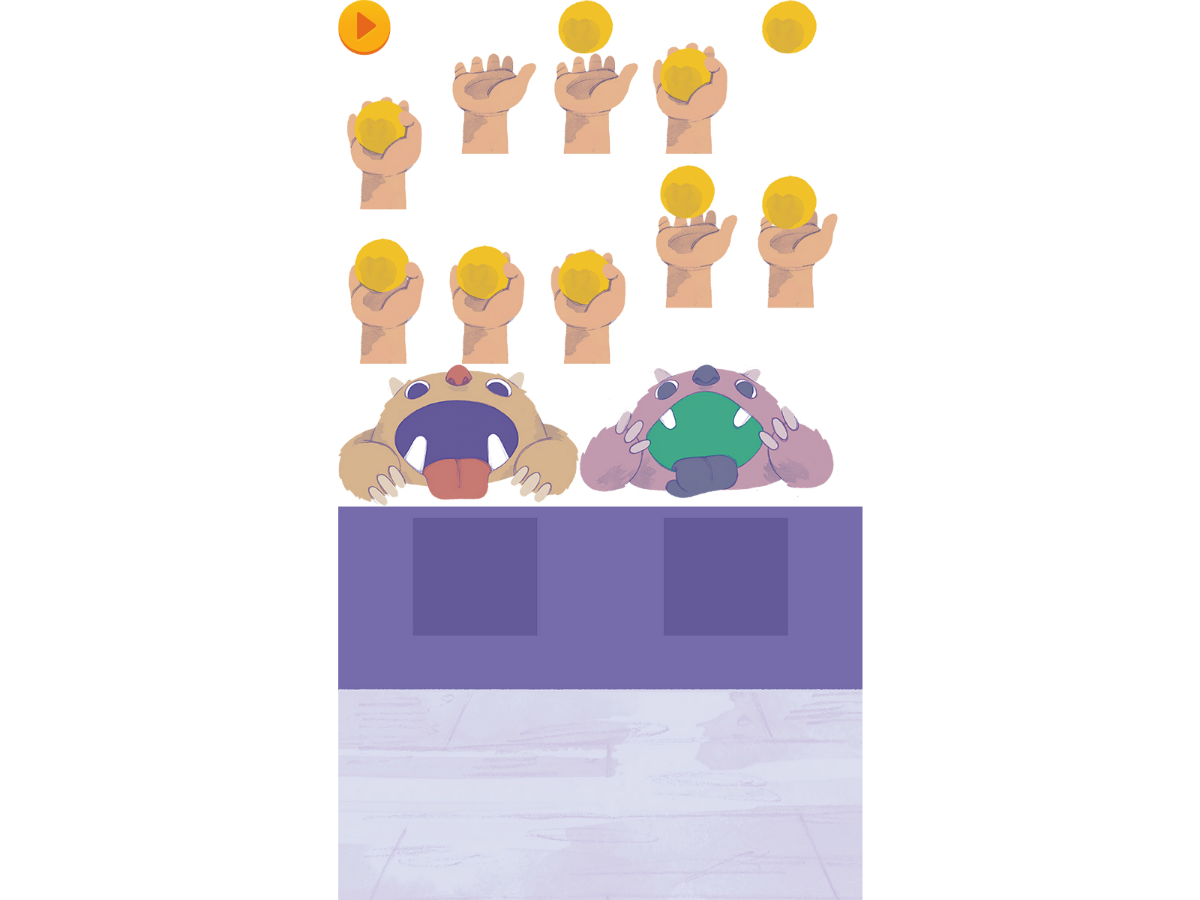
Sprite sheet sub for the Munching Monsters activity.

Care with expensive processes or requests have been addressed. For example, to avoid several requests to a server (for the online version) we opted to use sprite sheets (collections of static 2D drawings that depict representative poses [50]) that condense figures and textures, as shown in Figure 2. As the development is multi-device ready, the maximum image size was carefully controlled. To be on the safe side, and according to existing metrics [51], we used images with a maximum dimension of 2048×2048 pixels, which a device with as little as 256 MB RAM can still use.

**Figure 3:**
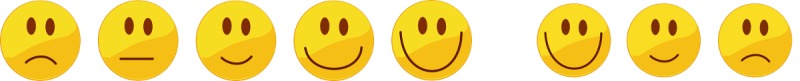
The “smileyometers”: Left (question 1 and question 2); right (question 3).

Since different screen dimensions had to be addressed, relative positioning of elements on screen using the dimensions of the Window in *innerWidth* (x) or *innerHeight* (y) as values was used. The *innerWidth* property returns the inner width of a window’s content area. The *innerHeight* property returns the inner height of a window’s content area. Due to different screen resolutions (eg, Apple Retina, Android Super Amoled, or liquid crystal display), one cannot assume a simple measurement of the window of the device as the size of the active area we can use as these screen technologies increase the number of pixels (or rather subpixels) per inch, improving the resolution. This results in different looks for the same activity, depending on the device characteristics, since a 300×300 pixels object in a device with a device pixel ratio (DPR) of 2 will look like it has 150×150 pixels. Therefore, “in game” scaling was implemented using a constant variable, which is the ratio of the vertical size of 1 pixel on the current display device to the size of 1 device independent pixel [52], divided by the highest DPR we expect the device to support [53].

### Beta Testing

Beta testing involves releasing and testing a software version with limited functionality to a group of the target users [54], without the participation of the developers in the test [55]. It can be divided into 5 stages [56]: (1) requirements analysis, (2) testing procedures, (3) reporting systems, (4) defect analysis and retesting, and (5) closure. Beta testing allows to extensively test the software, find bugs, and collect requirements and suggestions of end users [57]. However, these design principles, originally formulated for adults, cannot be scaled down for children due to their own particular needs and goals, which are not necessarily met by tools designed for adults [58]. Therefore, an adapted (in terms of their procedures and reporting systems steps) beta test approach was used to obtain feedback from the users.

We selected qualified participants (who have the characteristics of target population), specified test procedures and schedules, and planned specific roles [56]. Despite having a hypothesis and an expected outcome, we could only ascertain their validity after a set of sessions with the children. The beta testers were 22 children. Their ages were between 42 months and 78 months. The *T2T* materials were tested in 12 weekly individual therapy sessions, 45 min each.

The equipment used in sessions was an Asus Memo Pad 8, with an 8-inch Wide Extended Graphics Array screen, 1 GB memory, Quad-core 1.33 GHz processor, and weighting approximately 320 grams. It was running the *T2T* software as a native offline app.

A similar data gathering approach to the one used during the second phase was used, with the SLT taking extensive notes during each session. Questionnaires based on “smileyometers” [59], as shown in Figure 3, were also used. The following questions were asked to the children: Did you like to play this game? (like factor); Was it fun? (fun factor); and Would you play it again? (play again factor). The possible answers to the first question were as follows: 1–I did not; 2–A little; 3–Liked, 4–Liked a lot; and 5–Loved it. For the second question, they were as follows: 1–No; 2–Not much fun; 3–Some fun; 4–Fun; and 5–A lot of fun. For the third question, children could answer 1–Yes; 2–Maybe; and 3–No.

Direct observation of the children was structured around 3 areas (what were we looking for, who did it, and how did we do it), that is, looking at how children behaved, annotating their interactions with the app, and how one could improve this interaction.

During this phase, several bugs were found and corrected, the interaction design was refined to better suit the users’ touch screen capabilities [60], and some illustrations and sound files were improved or recreated. Sound was also exported from the original uncompressed .wav files as .mp3 and .ogg, to cover both Android [61] and Apple [62] operating systems.

Special care was devoted to the audio quality, since adherence to therapy is influenced by audio feedback, and model speech sounds have been shown to be a requirement in speech and language therapy [34]. Sound recordings took place in a cabin, produced by Absorsor, Portugal, with sound reduction of 45dB, located at University of Aveiro’s Speech, Language and Hearing Laboratory. Speech samples used for auditory feedback were all based on audio recordings of the same certified SLT (the third author of this paper), involved in *T2T* software development. The participant was sitting comfortably at a distance of about 30 cm, in front of an MKH20-P48 omnidirectional condenser microphone (Sennheiser, Germany) connected to a Scarlett 6i6 audio interface (Focusrite, UK) using a Gold Edition XLR Microphone cable (Mogami, USA). The recordings were made with Adobe Audition 3.0, at a sampling rate of 48,000 Hz, with 16 bits per sample, using the Focusrite Universal Serial Bus 2.0 ASIO Driver Audio Driver 1.8. The data were recorded in mono format .wav (Windows PCM) without compression. Raw audio recordings were manually segmented into around 955 individual .wav files with *Audacity 2.1.2* (Audacity Team).

## Results

### Activities Development Phase

**Figure 4:**
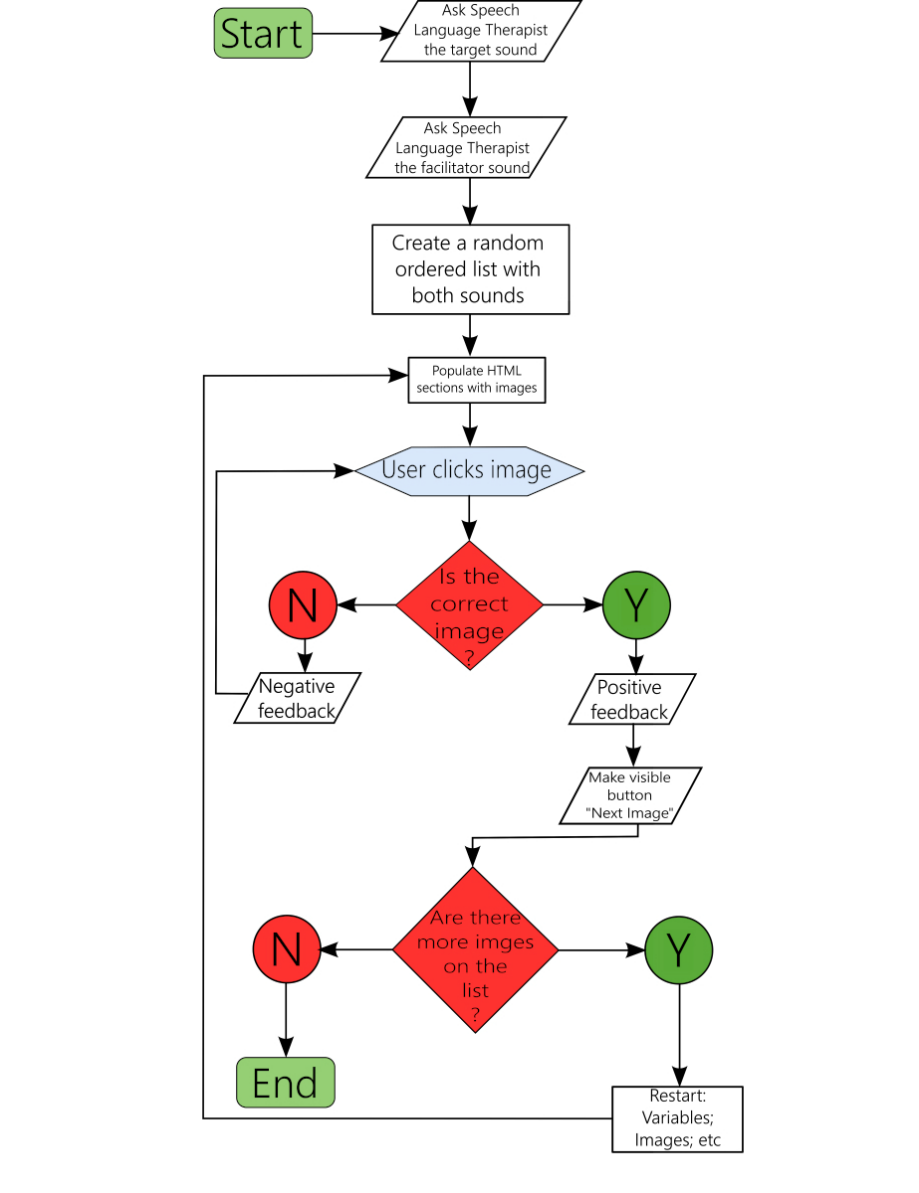
Simplified flowchart. SLT: speech and language therapist.

Flowcharts (see Figure 4) depicting the thought/activity process that resulted from the direct cooperation between the 6 members of the research team were initially produced for all activity areas. These flowcharts were the basis for the first version all computer-based activities, which then entered the beta testing phase.

**Figure 5:**
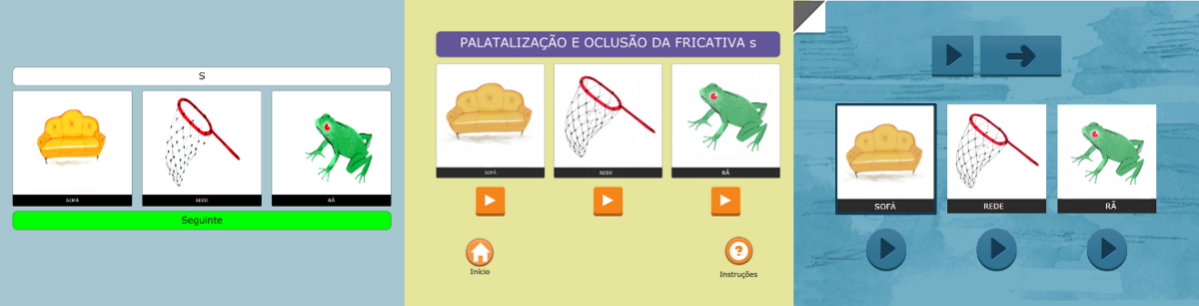
The evolution of the Match activity from the end of second development phase to beta testing. From less buttons and more dependent on the speech and language therapist (left) to less clutter on screen, bigger buttons to accommodate users with less touch screen capacities (middle), and more interactivity in terms of sound production and audiovisual feedback when the user completes an action (right).

Figure 5 presents an example of the first level of 1 activity of the grapheme-phoneme correspondence area. In this activity, the child must associate the grapheme to its sound in the word: the letter <S> has to be associated with the sound produced at the beginning of the word <sofa> (“couch”), excluding options available in the other 2 pictures. The child will then have a visual and audio feedback. Figure 5 also shows how the app evolved for this activity.

**Figure 6:**
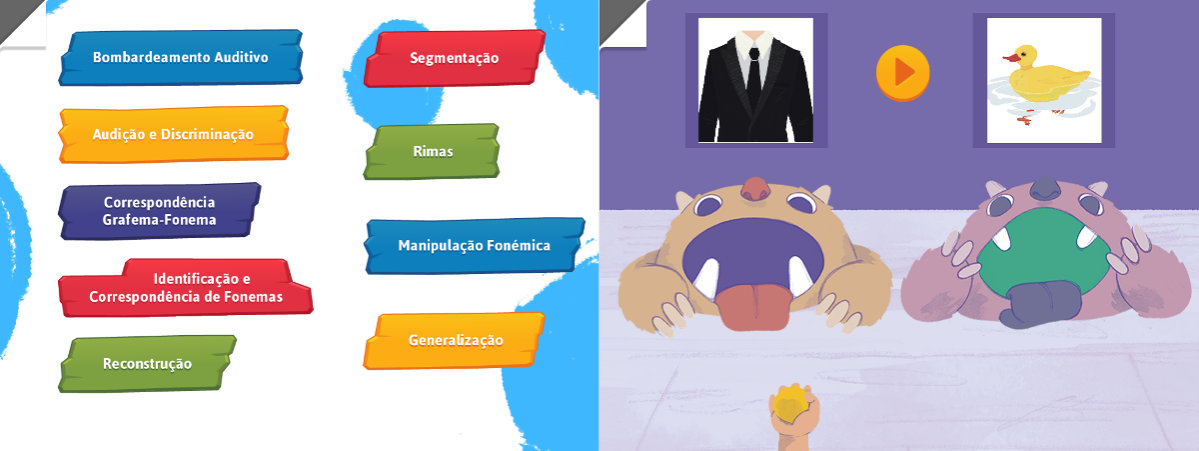
The main menu (left) and the Munching Monsters activity (right): area: hearing and discrimination; name: Munching Monsters; description: The child is presented with 2 open-mouth monsters each associated with an illustration of a minimal pair. At the bottom of the screen, there is a hand with a ball moving sideways. When “Play” is pressed, 1 of 2 possible words is heard. The child has to identify the corresponding image and release the ball with the right timing (into the mouth of the monsters). Digital audio feedback is given.

The main menu of the app (shown in Figure 6) shows a list of 9 areas of activities. A help area (mimicking a bent sheet of paper corner) is always present on the same location, independent of the screen or the area chosen. Inside it, there is a button to return to the home screen and a button to close the help area and resume the activity. The information presented is contextual. When the user chooses an area, 1 to 4 different activities might be found inside the area. Most of these activities have 2 difficulty levels, depending on the existence (level 1) or not (level 2) of a written word along with the recorded sound. The user first chooses 1 activity and then the difficulty level and is taken into a different screen with 9 possible problems (phonological processes) that can be targeted during the activity. After choosing the phonological process in which they aim to intervene, they go to a last panel before the actual activity (an example of an activity is shown in Figure 6). In this panel, they see a visual summary of their choices so far and can select the facilitating phonemes, minimal-pairs words, or rhymes, depending on the activity. Facilitating phonemes are those that the children can produce and should be able to differentiate. This allows an extensive customization of the activities. In this page, as well as in the previous pages, they always have the possibility of going back one step or back to the home screen.

After completing the process referred above, the activity starts (see Figure 6) and the SLT can intervene with the child. After a preset number of times, the activity will stop and the app returns to the home screen. During play, the SLT can read the instructions of the activity, can interact freely with the child, or rely on the app to produce most of the verbal feedback. The sounds can be played as many times as the child or the SLT deem necessary, and moving on to the next set of stimuli depends on the completion of the task.

### Ethnographic Pretest and Software Development

The activities’ development phase produced 19 activities (see Figure 5 for an example of an activity resulting from this phase); the ethnographic phase involved 7 testers; the software development phase included the activities’ development for mobile and desktop (Web) environments; and during the beta-test approach, phase extensive testing and reformulation was supported by direct, nonparticipant observation and a questionnaire adapted to children (with “smileyometer”).

### Beta Testing

Feedback regarding the activities was registered as part of the beta testing procedures of defect analysis and retesting. Detailed results for the tested activities are presented in Table 1. Due to the iterative nature of the method used, at the time of testing, 4 activities were in the process of being redesigned; therefore, no feedback from the original group of children was collected.

**Table 1:** Children’s feedback. The “smileyometers” have been converted to a 5-point Likert scale (question 1 and question 2)–possible integer values ranging from 1 to 5 and a 3-point Likert scale (question 3)–possible integer values ranging from 1 to 3.

Activity	Like, mean (SD)	Fun, mean (SD)	Play again, mean (SD)
Phoneme Tales	4.29 (1.03)	3.71 (1.28)	1.57 (0.73)
Listen and Build	4.86 (0.35)	4.43 (0.73)	1.00 (0.00)
Let’s Throw the Ball	5.00 (0.00)	4.43 (0.73)	1.00 (0.00)
Munching Monsters	5.00 (0.00)	4.86 (035)	1.00 (0.00)
Choose Well	4.43 (0.49)	4.43 (0.73)	1.14 (0.35)
Let’s Fish	4.86 (0.35)	4.86 (0.35)	1.00 (0.00)
Colouring Time 1	4.14 (1.12)	4.00 (1.07)	1.43 (0.49)
Colouring Time 2	4.29 (0.88)	4.14 (0.83)	1.57 (0.90)
Match	4.43 (0.73)	4.43 (0.64)	1.14 (0.49)
The Hungry Monster	4.43 (0.73)	4.14 (0.64)	1.43 (0.49)
­­ You Have Mail	5.00 (0.00)	4.71 (0.45)	1.00 (0.00)
­­ Find the Pairs	4.71 (0.45)	4.43 (0.49)	1.14 (0.35)
­­ Blend and Discover	3.71 (0.88)	3.57 (0.90)	1.14 (0.35)
­­ Sweet Tooth Bear	4.71 (0.45)	4.29 (0.88)	1.14 (0.35)

They were, however, tested later on, with a distinct group of children. We believe this would have introduced an additional confounding factor; therefore, we only report results from the same group. Moreover, 1 activity was not tested (the nineteenth activity) because it results in simple “yes-no” answers. Children are shown images (that before intervention they had difficulties in discriminating), and they should be able to produce the correct word elicited by them. If not, it is likely that further therapy is needed.

The activity *Colouring Time 1* was readjusted due to children’s lack of motivation to paint large areas. Results show that, overall, children “Liked a lot” all the activities except the *Blend and Discover* with a mean score of 3.71 (the lowest score of all). It should be noted that this activity is one of the least ludic so this factor could influence the children’s feedback. Regarding the fun factor, children’s overall feedback was “Fun” except for 2 activities: *Phoneme Tales* and *Blend and Discover*. Concerning the replay value (play again factor), there were 10 activities children would play again and 4 that did not present the same unanimity of feedback. *Phoneme Tales* and *Coloring Time 2* presented the least replay value. The similarity between *Coloring Time 1* and *Coloring Time 2*, and the fact that some children did not enjoy painting might have skewed our results.

High levels of satisfaction (question 1 average of 4.6 and SD of 0.5) were observed across the activities, with children liking the activities and finding them fun (question 2 average of 4.3 and SD of 0.7). When asked if they wanted to play them again (question 3 average of 1.2 and SD of 0.3), the result was yes. When combining the results of the Likert scale plus the direct observation in a qualitative fashion, the team was able to perceive some areas of enhancement.

Feedback was used to improve the software and interaction of the users with it [59]. All the code and design (graphic user interface and other elements) went through several iterations, constant optimization, and improvement over the years, and the joint team approach plus the constant data gathering improved several key areas of the app and/or the activities. For example, an activity called *Munching Monsters* had 3 major changes. The first design consisted of 2 pyramids of cans, each with an associated word that the child could try to knock over with a ball. The cans falling on the ground, the commotion, and noise generated proved (as a physical activity) to be too distracting. It was reformulated to 2 open-mouthed monsters, each with an associated word, and the child had to try to throw the ball into the monster’s mouth. As a digital app, a hand would be moving from left to right continuously, holding a ball, as shown in Figure 2. After hearing the word to discriminate, the child would tap the corresponding monster to throw the ball. It was observed that the child would try to do a sliding motion toward the monster or tap the hand. The final revision changed the behavior to tapping on the hand.

## Discussion

### Principal Findings

Different professionals, such as SLTs, kindergarten teachers, or psychologists, need materials that support their interventions. The most common type of materials is still pencil and paper, or card and board games–based, but in an era of technology, it is increasingly common to use tablets and other digital media. However, there are still few apps valid and adapted for languages other than English that allow intervention.

The *T2T* materials have been tested, built, and scientifically validated by a team with great expertise in the areas of speech and hearing sciences, mobile app development, and illustration. The use of *T2T*-based intervention programs by SLTs will allow these professionals to make a better and more effective communication intervention, based on proven methodologies that coexist in a structured physical and a digital version. These versions provide a full, 6-week intervention program, with minimal effort in preparing the session by the SLTs while delivering a very consistent intervention, with high replay value (as can be seen in Table 1).

A continuous communication and joint team approach was beneficial to the project and to the development of a solution focused on the real needs of SLTs and children with SSD. All problems were approached as a team with different skills and expertise, which minimized errors (eg, the developer making choices that would save him from spending time doing something that would not be used), and time spent. In addition, the importance of integrating the end users as testers and collecting their opinions and actions per session allowed the production of better-targeted activities.

The fact that the “smileyometer” scale used was not balanced in terms of presenting more perceived smiling faces than frowning ones might have biased the results. This feedback-gathering strategy should have been used on a larger sample of children. As the scale was applied by the therapist, it might have influenced the results.

The Web-based approach that allows to write the software once and deploy it across multiple operating systems and devices minimizes time and resources spent while facilitating the use of a natural interaction by the users with a touch capable device. Tablet-based therapy has an added benefit of portability, and the *T2T* app reduces the time spent preparing the sessions, translating in more time for the children.

### Future Work

A Web-based survey that has just been completed in Portugal using snowball sampling, showed that 96% of the total sample (N=101 that corresponds to 5% of the Portuguese SLTs) wanted to have more speech and language therapy apps available. In total, 56% (57/101) of respondents were Android users, 30% were iOS users, and 12% windows users, with the remaining 2% using more than 1 operating system. To meet this need, a new start-up company that aims to study, create, develop, and validate digital support materials for professionals working with communication is currently being developed. The aim is to develop specialized, economical, portable, multiplatform material that is able to work online and offline. The core product of this company will be the *T2T* software that is ready for commercialization, having been validated with a group of children proving its effectiveness.

Future studies that gather qualitative feedback regarding user experience and user interaction should rely on someone else besides the therapist to collect the data. The method proposed in this paper could be applied to other activities and materials not yet tested, such as traditional speech and language therapy physical materials or digital educational apps currently available.

Specifically concerning the *T2T* app, future research will improve interaction and functionality of the software, with more languages being offered and the creation of logs with scores and assorted data deemed necessary for the SLTs to better document a child’s evolution throughout intervention. Children’s feedback in designing new activities should continue to be encouraged. The creation of homework with gamification aspects that can appeal to a child to play and learn, while sending data to the SLTs in a secure way, has been considered as a much-needed companion to the *T2T* software. The first prototypes of 4 games are currently being tested.

## Abbreviations

DBR: design-based research
DPR: device pixel ratio
SLT: speech and language therapist
SSD: speech sound disorders
T2T: Table to Tablet
